# *De novo* Assembly and Characterization of the Testis Transcriptome and Development of EST-SSR Markers in the Cockroach *Periplaneta americana*

**DOI:** 10.1038/srep11144

**Published:** 2015-06-05

**Authors:** Wan Chen, Yu-Xiang Liu, Guo-Fang Jiang

**Affiliations:** 1 Jiangsu Key Laboratory for Biodiversity and Biotechnology, College of Life Sciences, Nanjing Normal University, Nanjing 210023, China

## Abstract

The cockroach *Periplaneta americana* is a notorious pest and threat to health worldwide, with a high reproductive ability. However, a limited amount of data is available on the developmental stage-specific transcriptomes of *P. americana*. To identify genes involved in developmental processes and to develop additional SSR markers in *P. americana*, we carried out *de novo* assembly of the *P. americana* transcriptome using Illumina sequencing. After removing low-quality sequences, we obtained 64,954,709 contigs, which were further assembled into 125,390 unigenes with an average length of 711 bp. Based on similarity searches against known proteins, we identified 48,300 unigenes based on a cut-off E-value of 10^−5^. The assembled sequences were annotated according to gene descriptions, gene ontology and clusters of orthologous groups. A total of 14,195 potential SSRs were identified, and 41 of 63 randomly chosen primer pairs successfully amplified the predicted SSR markers, seven of which were polymorphic in size in *P. americana*. Furthermore, the *Spag6* gene was confirmed to be testes specific, and the *fru* and *RPSA g*enes were related to the development of the testis. This is the special report of a *P. americana* transcriptome obtained using Illumina sequencing technology, and a large number of molecular markers were developed.

The cockroach is one of the oldest known winged insects, and its habitats are closely associated with those of humans. To date, over four thousand species of cockroaches have been identified, approximately 30 of which are harmful to humans^1^. *Periplaneta americana* (Linnaeus) is the most common domestic species of cockroach in the world and shows an extremely high reproductive capability. *P. americana* has been used as a model organism to study the effects of adipokinetic hormones^2^, sexually dimorphic glomeruli and related interneurons^3^, and apoptosis in the midgut nidi^4^. However, the developmental and reproductive processes of *P. americana* have not been well studied, however such studies are vital for the biological control of the species. Moreover, insufficient genomic information is available for *P. americana*, limiting our understanding of the molecular mechanisms underlying the developmental and reproductive processes of *P. americana*.

Over the past several years, next-generation sequencing technologies, such as the Roche 454, Illumina Solexa GA and ABI SOLID platforms, have emerged as cutting-edge approaches for high-throughput sequencing^5^, which dramatically improves the efficiency of gene discovery^6^,^7^. Furthermore, next-generation sequencing has significantly improved the sensitivity of gene expression profiling and contributed to comparative genomic studies^8^,^9^. Illumina sequencing of the transcriptomes of organisms with completed genomes previously confirmed that relatively short reads can be effectively assembled and used for gene discovery and comparison of gene expression profiles^10^,^11^. For non-model organisms with limited genomic information, transcriptome sequencing is a cost-effective tool because it focuses mostly on the sequencing of functional and protein-coding RNAs^12^. Despite the feasibility of this methodology, Illumina next-generation sequencing has not yet been applied to *P. americana*.

Transcriptome sequencing is an effective method for obtaining EST sequences, which are essential for developing molecular markers and identifying novel genes. We focus on two types of markers, simple sequence repeats (SSRs) and single-nucleotide polymorphisms (SNPs). SSRs have been widely used in studies for gene identification and fingerprint mapping because they exhibit high levels of polymorphism and are associated with simple protocols and high reproducibility. Currently, only a few SSR primers have been developed in *P. americana* because the standard methods for developing SSR markers are time-consuming and expensive. Deep transcriptome sequencing provides a good resource for the development of SSRs because of its high throughput. Another type of marker, SNPs, are the most abundant type of marker and can be easily detected via high-throughput sequencing, which will be helpful in future linkage and associated studies.

Using transcriptome data, we closely examined several candidate genes involved in mating in males. For example, the Sperm-associated Antigen 6 (*Spag6*) gene was confirmed to be testes specific, and the *fruitless* (*fru*) and *RPSA g*enes were related to the development of the testis. *Spag6* is essential for flagellar motility and maintenance of the structure of the axoneme of mature sperm in mice^13^. *Spag6* may play similar roles in testicular function in *P. americana*. Another gene, *fru*, appears to be the master regulator affecting male courtship behaviours. These courtship behaviours are either abnormal or absent in male flies lacking the *fru* gene, and the male-specific variants of *fru* are necessary and sufficient to elicit male courtship behavior^14^. This function is also very likely to be conserved in *Blattella germanica*^15^.

In this study, we generated over six billion bases of high-quality DNA sequences through Illumina sequencing and confirmed the suitability of using short-read sequencing for the *de novo* assembly and annotation of genes expressed in a eukaryote without reference genome information.

## Results

### Illumina sequencing and read assembly

cDNA samples were prepared from the testes of adult males of *P. americana* and sequenced using Illumina sequencing. After cleaning and quality checks, we obtained 6.3 Gb of reads. To facilitate sequence assembly, these raw reads were randomly clipped into 25-mers for sequence assembly using Trinity software^16^. These short 25-mers were subsequently assembled, resulting in 64,954,709 contigs, which were further assembled into 125,390 unigenes with an average length of 711 bp, ranging from 351 bp to 21,092 bp, including 24,887 unigenes larger than 1,000 bp (Table 1). To double check the quality of the sequencing data, we randomly selected 10 unigenes and designed 10 primer pairs for RT-PCR amplification. Amplification resulted in the expected product size in 8 of the 10 unigenes, and the sequences of all eight PCR products were confirmed using Sanger sequencing (data not shown).

### Annotation of predicted proteins

**Figure 1:**
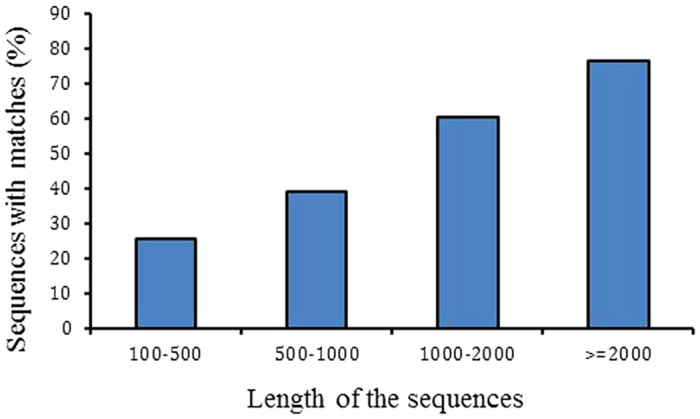
The proportion of sequences with significant matches increases with query sequence length. The proportion of sequences with matches (with a cut-off E-value of 1.0E^−5^) in NCBI nr databases is significantly greater among the longer assembled sequences.

**Figure 2:**
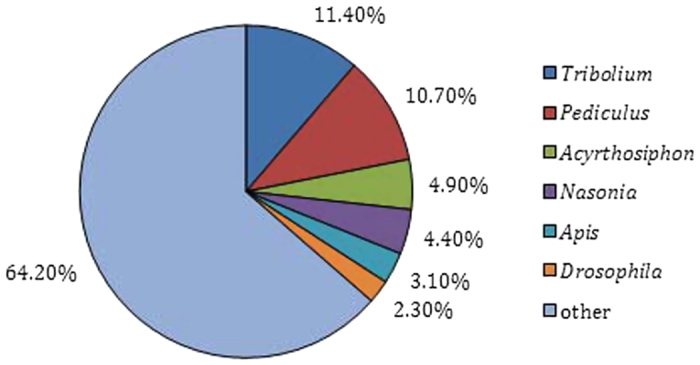
Species distribution for homology search of Illumina sequences against the nr database. Species distribution is shown as percentage of the total number of homologous sequences with an E-value cutoff of 1.0E^−5^. The most significant hit of each sequence was used for further analysis.

For annotation, the unique gene sequences were first subjected to BLAST searches against the non-redundant NCBI nucleotide database (nr) employing Blastx with a cut-off E-value of 10^−5^. Using this approach, 48,300 unigenes (38.5% of all distinct sequences) returned a BLAST hit above the cut-off. These annotated unigenes formed a potential pool for gene identification in *P. americana*. Because of the relatively short length of the query sequences and the lack of genome information for cockroaches, most of the 77,090 assembled transcripts (61.5%) could not be matched to known genes. The proportion of sequences showing hits in nr databases was higher among the longer assembled sequences. Specifically, 76.3% of assembled sequences longer than 2,000 bp returned significant BLAST hits, whereas the proportion decreased to 60.5% for sequences ranging from 1,000 to 2,000 bp, to 38.9% for those between 500 and 1,000 bp and to 25.6% for sequences of less than 500 bp (Fig. 1). Regarding the species distribution, 11.4% of the distinct sequences showed top matches with sequences from the red flour beetle (*Tribolium castaneum*), followed by the body louse (*Pediculus humanus corporis*) (10.7%), the pea aphid (*Acyrthosiphon pisum*) (4.9%), and the jewel wasp (*Nasonia vitripennis*) (4.4%) (Fig. 2). Given the scarcity of prior information from *P. americana*, only 697 unigene sequences (0.56%) were matched with the highest degree of homology to genes from *P. americana*, and the majority of these hits were matched to lectin-related proteins (data not shown).

### Gene ontology (GO) classification

**Figure 3:**
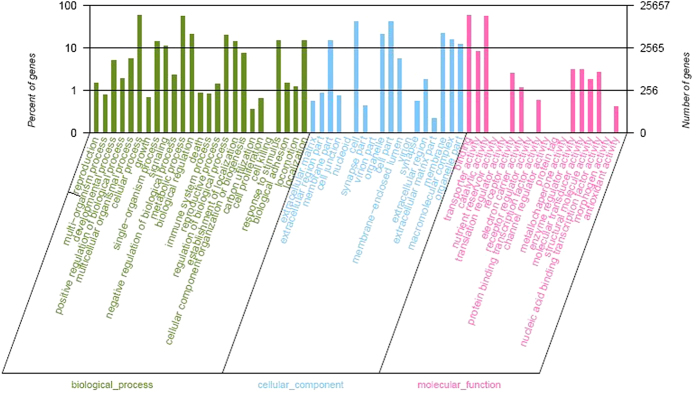
Histogram presentation of Gene Ontology classification. Results are summarized in three main categories: biological process, cellular component and molecular function. The left y-axis indicates the percentage of a specific category of genes in that main category. The right y-axis indicates the actual number of genes in a category.

Based on the nr annotation, GO assignments were employed to classify the functions of the *P. americana* transcripts. Among the 48,300 nr hits, a total of 25,661 sequences could be categorized into 61 functional groups (Fig. 3). Within the three main categories (biological process, cellular component and molecular function) of the GO classification, the 'Cellular process', 'Cell part' and 'Binding' terms were most prevalent, respectively. We also noted that a high percentage of genes were classified under the 'Metabolic process', 'Cell' and 'Catalytic activity' terms, while only a few genes were classified under the terms 'Cell killing', 'Virion part' and 'Morphogen activity' (Fig. 3).

### Clusters of orthologous groups (COG) classification

**Figure 4:**
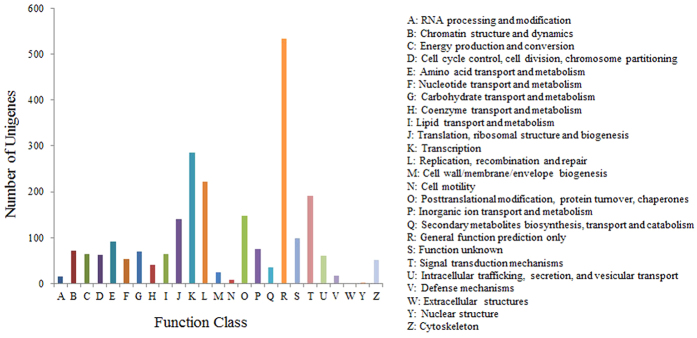
Histogram presentation of clusters of orthologous groups (COG) classification. Out of 48,300 nr hits, 3,112 sequences have a COG classification among the 25 categories.

In total, 3,112 of the 48,300 nr hits showed a COG classification (Fig. 4). Among the 25 COG categories, the cluster for 'General function prediction' represented the largest group (534, 17.2%), followed by 'Transcription' (285, 9.2%) and 'Replication, recombination and repair' (222, 7.1%). However, we did not find any genes under the 'Extracellular structures' category. The following categories represented the smallest groups: Nuclear structure (1, 0.03%), Cell motility (8, 0.26%) and RNA processing and modification (16, 0.51%) (Fig. 4).

### Functional classification using Kyoto Encyclopedia of Genes and Genomes (KEGG)

All of the unigenes were analysed in the KEGG Pathway database, and 21,768 (17.4%) unigenes showing significant matches in the database were assigned to 312 KEGG pathways. Among these pathways, 'metabolic pathways' constituted the largest category (3,929, 18%), followed by 'biosynthesis of secondary metabolites' (864, 4%), 'Purine metabolism' (676, 3.1%) and 'pathways in cancer' (569, 2.6%).

### SNP detection

A total of 1,379 high-quality SNPs were identified among all of the unigenes (Table 2). In the quality filtering steps, the filtered SNPs were required to have at least 150 bp of flanking sequence on both sides of the SNP for further analysis. The predicted SNPs included 581 transitions (298 C/T and 283 A/G transitions) and 798 transversions (224 A/T, 160 A/C, 166 T/G and 248 C/G transversions).

### Development and characterization of SSR markers

To evaluate the quality of the assembly and develop new molecular markers, 125,390 unigenes assembled in this study were used to mine potential SSRs. The potential SSRs were defined as ranging from mononucleotide motifs with a minimum of ten repeats to hexanucleotide motifs with a minimum of three repeats. A total of 14,195 potential SSRs were identified, which could be divided into two types of mononucleotide SSRs, four types of dinucleotide SSRs, ten types of trinucleotide SSRs, twenty-nine types of tetranucleotide SSRs, thirty-four types of pentanucleotide SSRs and six types of hexanucleotide SSRs. The largest fraction of identified SSRs consisted of mononucleotides, which accounted for 59.8% (8,488) of the SSRs. The second most common type of SSR was trinucleotides, accounting for 26.9% (3,817) of the SSRs, followed by dinucleotides (1,190, 8.4%), tetranucleotides (633, 4.5%), pentanucleotides (61, 0.4%) and hexanucleotides (6, 0.04%). Among the identified SSRs, A (T) (57.1%) accounted for 95.5% of the mononucleotide repeats, and AC (GT) (3.3%) accounted for 39.1% of the dinucleotide repeats, while AAT (ATT) (8.1%), ATC (GAT) (5.5%) and AAG (CTT) (3.9%) together accounted for 65.5% of the trinucleotide repeats (Table 3).

To verify the identified SSR markers, we attempted to amplify the predicted SSRs via PCR. BatchPrime3 was used to design appropriate primers based on the flanking sequences. A total of 1,292 primer pairs for 1,186 out of 3,756 SSRs were successfully designed, while the remaining SSRs were not suitable for primer design. 63 primer pairs were randomly selected to amplify the genomic DNA of *P. americana*. Among the 63 primer pairs, 41 resulted in successful PCR amplification, whereas the remaining 12 primer pairs failed to generate PCR products at various annealing temperatures. Among the 41 successfully generated PCR products, 32 showed specific amplification, with 26 of these products presented the correct size, while five were longer than expected, and one primer pair amplified a significantly shorter product than expected.

Two *P. americana* clusters, including 10 individuals from Nanjing (Jiangsu province) and 10 from Taizhou (Zhejiang province), were randomly selected for amplification and polymorphism analysis. PCR amplification was performed according to the method of Deng *et al.*^17^. The 26 primer pairs that resulted in correct amplification were further used to help evaluate polymorphism levels in these two *P. americana* clusters. Eight primer pairs amplified products of a single size, while the other 18 primer pairs amplified polymorphic products. Seven of the 18 primer pairs amplified products of the expected size in all *P. americana* clusters, while 11 primer pairs generated longer PCR products than expected. For the seven polymorphic loci, there were 2–3 alleles at each locus. The observed heterozygosity (*Ho*) varied from 0.3333 to 0.8333 (0.5 ± 0.1925), while the expected heterozygosity (*He*) varied from 0.3182 to 0.803 (0.5476 ± 0.1881). The polymorphism information content (PIC) values ranged from 0.2723 to 0.6874 (0.4389 ± 0.1718) (Table 4).

### Genes related to the development of the testis

In the gene ontology (GO) classification, we focused on the 'Reproduction' term in the biological process category. A total of 386 unigenes were assigned to the 'Reproduction' term, which are likely to participate in reproductive processes. A further Blastx research was performed for these 386 unigenes, and scores above 80 for Blastx and a query cover above 30% were considered significant^1^. Among the remaining 261 unigenes (Table S1), the Sperm-associated Antigen 6 (*Spag6*) and *fruitless* (*fru*) genes were confirmed to be testes specific in previous studies. qRT-PCR was carried out to verify whether these two genes were also testes specific in *P. americana*. The primers used to amplify these two genes are listed in Table 5.

**Figure 5:**
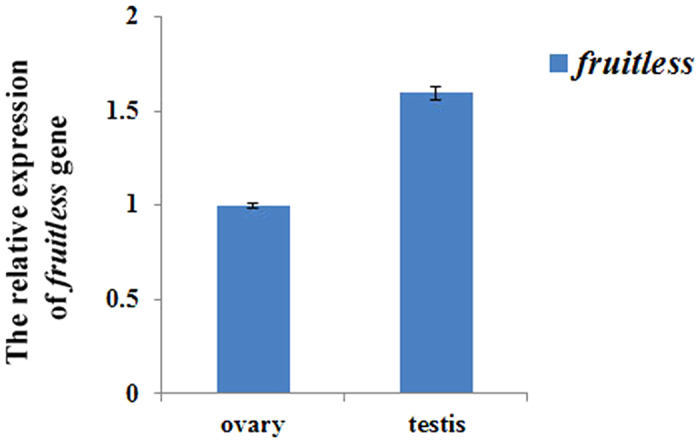
Phylogenetic analysis of RPSA in insect species. Neighbor-joining method was used to construct the phylogenetic tree. Bootstrap values with 1000 trials are indicated on branches.

**Figure 6:**
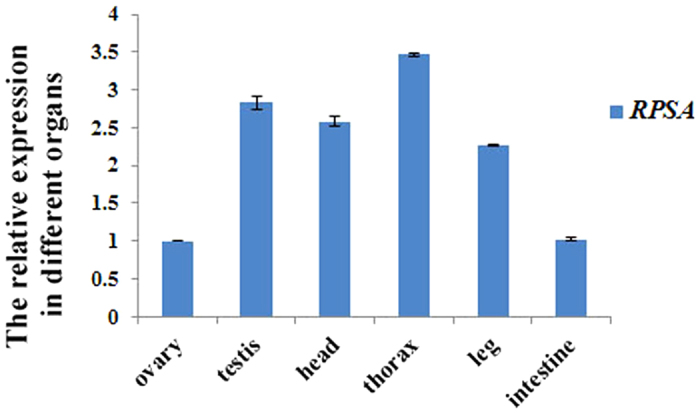
Quantification of the expression level of fruitless gene with real-time PCR. The expression level of this gene in female *P. americana* was set as 1. All the data collected from the real-time PCR analyses were shown as averages ± SE; P < 0.01.

The RT-PCR results showed that the *Spag6* gene was expressed only in the testis of male *P. americana*, and not in the ovary of females. The qRT-PCR results revealed that *fru* transcripts were approximately 1.6-fold more abundant in male *P. americana* than that in females (Fig. 5). Real-time PCR showed that *RPSA* was expressed in almost all of the tissues of *P. americana* (Fig. 6), presenting the highest expression level in the thorax, followed by the testis, head and leg, whereas it exhibited a low expression level in the ovary and intestine.

## Discussion

To determine the gene expression profile in the testes of adult male *P. americana*, cDNA samples were prepared from the testes of adult males and sequenced through Illumina sequencing. After cleaning and quality checks, we obtained 6.3 Gb of reads. The genome size of *P. americana* was 3,338 Mb^18^, and approximately 6.3 Gb of transcriptome sequence data likely represented 1.9-fold coverage of the genome of *P. americana.* The *de novo* assembly of lower eukaryotic transcriptomes is straightforward, with more than 30-fold coverage being required for the full length of most yeast transcripts, whereas the *de novo* assembly of higher eukaryotic transcriptomes is much more challenging because of the larger sizes of the datasets and the difficulties involved in identifying alternatively spliced variants^5^,^19^,^20^. In spite of the limitation in terms of coverage, a large set of *P. americana* transcripts can provide valuable information for further gene identification and the identification of molecular markers. To facilitate sequence assembly, these raw reads were further assembled into 25,390 unigenes with an average length of 711 bp.

The KEGG Pathway database is a collection of manually drawn pathway maps representing current knowledge regarding molecular interaction and reaction networks. Pathway-based analysis is helpful to better understand biological functions and gene interactions. Among the 312 KEGG pathways to which the obtained unigenes were assigned in this work, 'metabolic pathways' represented the largest category (3,929, 18%), followed by 'biosynthesis of secondary metabolites' (864, 4%), 'PURINE METABOLISM' (676, 3.1%) and 'pathways in cancer' (569, 2.6%). These results imply that active metabolic processes were underway in the testes^21^. The KEGG functional classification provided a valuable resource for investigating specific processes, functions and pathways in the testes of *P. americana*.

Genetic markers are important for studying population structure, diversity and the genetic basis of adaptive traits^22^,^23^. Markers based on transcriptome sequences are useful for the detection of variation and functional genetic analysis^24^. To verify the identified SSR markers, we attempted to amplify the predicted SSRs via PCR. A total of 1,292 primer pairs for 1,186 out of 3,756 SSRs were successfully designed using BatchPrime3, while the remaining SSRs were not suitable for primer design. For these SSRs, no appropriate primer pairs could be found for the following reasons: (1) the SSRs were located too close to the end of the flanking region, or (2) the base composition of the flanking sequence was unsuitable. In our results, the observed Ho value varied from 0.3333 to 0.8333 (0.5 ± 0.1925), while the expected He varied from 0.3182 to 0.803 (0.5476 ± 0.1881). PIC values ranged from 0.2723 to 0.6874 (0.4389 ± 0.1718) (Table 4). These results suggested that the primers designed to amplify SSR loci could serve as tools for polymorphism evaluation in *P. americana*. Furthermore, the predicted SSR loci could serve as a useful resource for SSR marker development.

In the Gene Ontology (GO) classification, we focused on the 'Reproduction' term in the biological process category. *Spag6* was confirmed to be a testes-specific gene, and the *fru* and *RPSA g*enes were related to the development of the testis. The results of RT-PCR showed that the *Spag6* gene was expressed only in the testis of male *P. americana*, and not in the ovary of females. *Spag6* is essential for sperm motility in mice, and a lack of *Spag6* leads to inviability or infertility^13^. We speculate that *Spag6* may play a similar role in controlling the testicular development of *P. americana*. Additionally, the qRT-PCR results showed that *fru* transcripts were approximately 1.6-fold more abundant in male *P. americana* than that in females, suggesting that the *fru* gene might play an important role in testicular development. In *Drosophila melanogaster*, more than 30 genes have been shown to affect behavioural steps in male courtship, and *fru* appears to be the master regulator. Male flies lacking the *fru* gene are sterile, with male-specific courtship behaviours being either abnormal or absent^14^, and the male-specific variants of *fru* are necessary and sufficient to elicit male courtship behaviour. A previous study on the German cockroach (*B. germanica*) showed that *fru* plays a similar role in controlling male sexual behaviour in this species. Therefore, this role has been conserved during insect evolution, at least from the phylogenetically basal order Blattaria to the most distal Diptera^15^. This function is also very likely to be conserved in *P. americana*.

*RPSA* has numerous known functions. It is a multifunctional protein that is present in several cellular compartments. In our GO classification, *RPSA* was assigned to the “Cell part” and “Macromolecular complex” terms. This receptor is an extracellular matrix glycoprotein that mediates cell attachment, movement, differentiation and growth^25^. It has also been associated with the regulation of cytoskeletal dynamics^26^ and is expressed in a wide variety of tissues, including showing expression in tumour cells^27^,^28^. In a previous study by our group^29^, the transcription level of *RPSA* in the testis of *P. americana* was found to be 2.83-fold higher than in the ovary, in accord with a previous study in which the non-normalized gene expression level of *RPSA* in the testis was 6.3-fold higher than in the ovary^30^. Therefore, we speculate that *RPSA* may play a greater functional role in the testes than in the ovaries. In a previous study, this protein was shown to be upregulated by testicular heat shock^31^, and we predict that it might be involved in regulating the process of capacitation necessary for fertilization. The real-time PCR results showed that *RPSA* was expressed almost in all tissues of *P. americana*. Interestingly, it exhibited the highest expression level in the thorax, followed by the testis, head and leg, whereas a low expression level was observed in the ovary and intestine. Hence, it is suggested that *RPSA* plays a more important role in the thorax than in other tissues. However, we are still a long way from understanding how a specific ribosomal protein gene might contribute to the development of the thorax as well as gonadal development. Although the function of *RPSA* in reproduction has not yet been reported, *RPSA* would be a candidate gene for further research on fertilization. Additional studies on the molecular mechanisms of this gene involved in controlling the development of the thorax and testis should be carried out to obtain a better understanding of the development of a number of the organs of *P. americana*.

The assembled, annotated transcriptome sequences and gene expression profiles can serve as an invaluable resource for future identification of *P. americana* genes involved in reproduction.

## Conclusion

In this study, we sequenced and analysed *P. americana* transcriptomes. A large-scale transcriptome dataset with 125,390 unigenes was produced, with 48,300 of these unigenes showing a BLAST hit below a cut-off E-value of 10^−5^. A total of 14,195 potential SSRs were identified, and 1,292 primer pairs were successfully designed. The SSR markers developed in this work constitute a valuable resource for studying genetic diversity, linkage mapping, and germplasm characterization analysis in cockroaches. The results confirmed that transcriptome analysis based on Illumina paired-end sequencing is a cost-effective and reliable approach in non-model organisms. In addition, the *Spag6* gene was confirmed to be testes specific in *P. americana* using RT-PCR, and the *fru* and *RPSA* genes were related to the development of the testis in *P. americana* via real-time PCR. The present study provides new clues towards elucidating the molecular mechanisms underlying gonad development in *P. americana*. The obtained transcriptome information will also serve as an important public dataset for further research on gene expression, genomics, and functional genomics in *P. americana*.

## Methods

### Insect rearing and sample preparation

The specimens of *P. americana* were obtained in our laboratory. The colony was maintained at 25 °C, 60–70% r.h., with a 12 h:12 h daily light:dark cycle. Cockroaches that had moulted to the adult stage twenty to thirty days earlier were selected. We collected the testes from 10 males. All dissections and tissue samplings were carried out in carbon dioxide-anaesthetized specimens^32^.

### RNA isolation and library preparation for transcriptome analysis

Total RNA was extracted from the head, thorax, legs, intestines, testes and ovaries of *P. americana* using the RNeasy® Plant Mini Kit (Qiagen, Hilden, Germany), following the manufacturer's protocols^32^. Considerable care was taken to ensure that all of the total RNA samples used in this study were of high quality (showing an A260/A280 > 1.8 in nuclease-free water), with minimal degradation^33^. Briefly, mRNA was purified from 5 μg of total RNA using oligo (dT) magnetic beads. Following purification, the mRNA was fragmented into small pieces using divalent cations under elevated temperature, and the cleaved RNA fragments were employed for first-strand cDNA synthesis using reverse transcriptase and random primers. This was followed by second-strand cDNA synthesis using DNA polymerase I and RNaseH. These cDNA fragments then underwent an end repair process and were ligated with adapters. These products were finally amplified via PCR to generate a cDNA library for sequencing.

### Analysis of the Illumina sequencing results

The cDNA library was sequenced on the Illumina HiSeq2000 sequencing platform. The raw reads were cleaned by removing adaptor sequences, empty reads and short sequences with a length of less than 25 nt. The obtained reads were randomly clipped into overlapping *K*-mers (default K = 25) for assembly using Trinity software^16^. Distinct sequences were subjected to BLAST searches and annotation against the NCBI nr database using an E-value cut-off of 10^−5^. The obtained functional annotations according to Gene Ontology terms (GO; http://www.geneontology.org) were analysed using Blast2GO software. COG annotation was performed using Blastx 2.2.24 + software against the STRING 9.0 database. KEGG pathway annotation was performed using Blastx/Blastp 2.2.24 + software against the Kyoto Encyclopedia of Genes and Genomes database. Potential SNPs were detected using Samtools (http://samtools.sourceforge.net/) and VarScan (http://varscan.sourceforge.net/).

### Quantitative RT-PCR expression analysis

The quantitative RT-PCR was performed using the Rotor-Gene Q real-time PCR system. The SYBR Premix Ex Taqkit (Takara) was employed according to the manufacturer’s protocol. In each amplification reaction, gene-specific primers were used; each primer set exhibited a unique optimal Tm in the real-time PCR assays. The *β-actin* rRNA gene was used as the internal reference, and the expression level of each gene was normalized against the *β-actin* rRNA gene. Melting curves were checked to ensure that the primers were amplifying the desired amplicons, and the obtained values reflected increases in the amplicons, rather than primer dimers or other unrelated sequences. The calculated efficiency values for the above genes and the *β-actin* rRNA gene amplicons were all within the range of 95 to 100%; therefore, no correction for efficiency was applied in further calculations. Relative expression values were calculated from three biological replicates using a modified 2^−∆∆CT^ method^34^. All of the data collected from the real-time PCR analyses are presented as averages ± SE.

### SNP detection

SNPs were inferred from sequence variations in mixed individual pools of *P. Americana* specimens. Samtools and VarScan v.2.2.7 were used to identify candidate SNPs.

### Development and detection of SSR markers

Msatcommander was employed to identify repetitive elements in the assembled transcriptome of *P. americana*^35^. We focused on EST-SSRs with motifs ranging from two to six nucleotides and a minimum of 3 contiguous repeat units. Primer sequences were designed using BatchPrimer3 based on the flanking sequences, and the following criteria were required: 1) EST-SSRs with a minimum of seven repeats for dinucleotide motifs, five for trinucleotides, four for tetranucleotides and three for penta- and hexanucleotides; 2) a primer length between 18–22 bp, with an optimum of 20 bp; 3) a GC content between 40–60%, with an optimum of 50%; 4) a Tm of 55–65 °C (with a difference of no greater than 4 °C between the Tm values of the forward and reverse primers); and 5) an amplicon length of 100–300 bp with no secondary structure; for other parameters, the default settings were used.

Genomic DNA was isolated from the legs of different *P. americana* individuals using the Wizard^TM^ Genomic DNA purification kit (Promega, USA) following the manufacturer’s protocol. A total of 63 primer pairs were designed to check the correctness of the randomly selected SSR loci. The PCR products were analysed via electrophoresis on 8.0% non-denaturing polyacrylamide gels with silver staining^36^.

The appearance of each band was recorded as present (coded as 1) or absent (coded as 0). The scored data from polymorphic loci were used to calculate the polymorphism information content (PIC) according to the formula PIC = 1-∑p_i_^2^ (where p_i_ is the frequency of the i^th^ allele at each locus)^37^. We used Popgene version 1.31 38 to calculate the observed heterozygosity (*Ho*) and expected heterozygosity (*He*)^39^,^40^,^41^,^42^,^43^,^44^,^45^.

## Additional Information

**Accession codes**: All of the raw RNA-Seq data have been submitted to the NCBI databases (http://trace.ncbi.nlm.nih.gov/Traces/sra/sra.cgi?view=run_browser) under accession number SRR1321695, but the RNA-Seq data have not yet been released. The full dataset is also available from Guo-Fang Jiang upon request (cnjgf1208@163.com).

**How to cite this article**: Chen, W. *et al*. De novo Assembly and Characterization of the Testis Transcriptome and Development of EST-SSR Markers in the Cockroach Periplaneta americana. *Sci. Rep.* 5, 11144; doi: 10.1038/srep11144 (2015).

## Supplementary Material

Supplementary Information

## Figures and Tables

**Table 1:** Summary for the *Periplaneta americana* testis transcriptome.

**Total number of reads**	**64,954,709**
**Total base pairs (bp)**	**6,307,223,923**
Total number of genes	72,505
Total number of unigenes	125,390
Transcriptome size	96,700,846
Average unigenes length (bp)	771
Longest unigenes (bp)	21,092
Shortest unigenes (bp)	351
Sequences with BLAST E-value < 10^−5^	48,300

*Total number of genes is defined as the number of genes with homology in other insects.

**Table 2:** Summary of SNPs identified from testis transcriptome of *Periplaneta Americana*.

**Type**	**Number of counts**
Transition	581
C/T	298
A/G	283
Transversion	798
A/T	224
A/C	160
T/G	166
C/G	248
Total	1379

**Table 3:** Summary of SSRs identified from the testis transcriptome of *Periplaneta Americana*.

**SSR type**	**Repeats**	**Total number**	**Proportion of total SSRs (%)**	**Longer than 15bp (counts)**
Mononucleotide	Total	8488	59.80	585
	A(T)	8107	57.11	580
	C(G)	381	2.68	5
Dinucleotide	Total	1190	8.38	486
	AC(GT)	465	3.28	212
	AG(CT)	375	2.64	151
	AT(AT)	340	2.40	122
	CG(CG)	10	0.07	1
Trinucleotide	Total	3817	26.89	1064
	AAC(GTT)	287	2.02	43
	AAG(CTT)	551	3.88	148
	AAT(ATT)	1147	8.08	416
	ACC(GGT)	253	1.78	46
	ACG(CGT)	69	0.49	11
	ACT(AGT)	186	1.31	46
	AGC(GCT)	277	1.95	42
	AGG(CCT)	140	0.99	14
	ATC(GAT)	803	5.66	295
	CCG(CGG)	104	0.73	3
Tetranucleotide	Total	633	4.46	633
Pentanucleotide	Total	61	0.43	61
Hexanucleotide	Total	6	0.04	6

**Table 4:** Characterization of polymorphic microsatellite loci in the *Periplaneta americana* testis transcriptome.

**Locus**	**Primer sequence (5’-3’)**	**Repeat motif**	**Size (bp)**	**Tm (˚C )**	***H*_*O*_**	***H*_*E*_**	**PIC**
CR9	AGAGTAGGATAGGATAGGGTGGA	(GTAGG)_5_	150	58	0.8333	0.8030	0.6874
	CGCAGAAAGTCAATTTTGTGAT						
CR18	AGAAAATAAAGCCTGGGCAAC	(CCT)_8_	149	58	0.5000	0.4091	0.3047
	CTGTGGGGGTCTCTGTTGAG						
CR20	AAAGGTAGGTTGTGGTGGTGA	(ATCG)_6_	163	58	0.3333	0.4848	0.3457
	CGTTTTCATGAGGAGGGAACT						
CR24	TTGGCTGCAAAACAGAAACTT	(AAGA)_5_	173	58	0.3333	0.3182	0.2723
	CTCGCCCCACCTACAAATTAC						
CR31	TCAAGCTTATTTTGCATTCTCG	(AG)_10_	183	58	0.5000	0.4091	0.3047
	TCGACAAATCAACGACAGAAA						
CR47	GATGTGACACTCGTTGCAGTG	(TGC)_7_	156	56	0.3333	0.6667	0.5355
	GGCTCAGAAGCCTCTCTCAGTA						
CR50	CAACTGGCTCTGGACTGAAAA	(GGT)_7_	145	56	0.6667	0.7424	0.6218
	GTGATTTGGTTAGCACCATGC						

**Table 5:** The primers of genes detected by real-time PCR analyses.

**Putative genes**	**Real-Time PCR primers (5’–3’)**
*β-actin*	Forward primer: TTACCACCACTGCCGAACGA
	Reverse primer: CCTCTGGACAACGGAACCTC
*Fruitless*	Forward primer: ACTACCATCGGCTGGAGTTGGCATTC
	Reverse primer: GGTGTCTTGAGAGGCTGGACCTTGTT
*Sperm-associated Antigen 6*	Forward primer: CCTGCCTCTTGAGTTTTGGA
	Reverse primer: GTATCAGCGTCGGCTCTTTG
*Ribosomal protein SA*	Forward primer: TCGTCAGATGTATTGTAGGC
	Reverse primer: AAGTGGGATGTGGTGGTAGA
