# Corrigendum: *De novo* Assembly and Characterization of the Testis Transcriptome and Development of EST-SSR Markers in the Cockroach *Periplaneta americana*

**DOI:** 10.1038/srep16547

**Published:** 2015-11-19

**Authors:** Wan Chen, Yu-Xiang Liu, Guo-Fang Jiang

Scientific Reports 5: Article number: 1114410.1038/srep11144; published online: 06052015; updated: 11192015

In this Article, the legend of Figure 5 and Figure 6 are incorrect:

In Figure 5:

**"Phylogenetic analysis of *RPSA* in insect species.**

Neighbor-joining method was used to construct the phylogenetic tree. Bootstrap values with 1000 trials are indicated on branches."

should read:

**"Quantification of the expression level of *fruitless* gene in ovary and testis of *P. americana* with real-time PCR.**

The expression level of this gene in ovary was set as 1. All the data collected from the real-time PCR analyses were shown as averages ± SE; P < 0.01"

In Figure 6:

**"Quantification of the expression level of *fruitless* gene with real-time PCR.**

The expression level of this gene in female *P. americana* was set as 1. All the data collected from the real-time PCR analyses were shown as averages ± SE; P < 0.01."

should read:

**"Quantification of the expression level of *RPSA* gene with real-time PCR.**

The expression level of this gene in female *P. americana* was set as 1. All the data collected from the real-time PCR analyses were shown as averages ± SE; P < 0.01."

