# Supplementary material for: De novo Assembly and Characterization of the Testis Transcriptome and Development of EST-SSR Markers in the Cockroach Periplaneta americana
Source: Sci Rep. 2015 Jun 5;5:11144. doi: 10.1038/srep11144 (PMC4457154; doi:10.1038/srep11144)
Supplement: Supplementary Information [file srep11144-s1.pdf]

De novo* Assembly and Characterization of the Testis Transcriptome and Development of EST-SSR Markers in the Cockroach *Periplaneta americana

Wan Chen, Yu-Xiang Liu & Guo-Fang Jiang*

Jiangsu Key Laboratory for Biodiversity and Biotechnology, College of Life Sciences, Nanjing Normal University, Nanjing 210023, China.

Table S1. The 261 genes related to the development of testis in *Periplaneta americana*

Sequence	Length	Gene similar to	Accession	Query cover	E-value
comp100186_c0_seq1	486	takeout/JHBP like protein	BAM18028.1	79%	9.00E-39
comp100186_c0_seq2	486	protein takeout precursor,	XP_002432614.1	59%	1.00E-19
comp100723_c0_seq3	682	Cysteine-rich motor neuron	EGW09830.1	99%	2.00E-23
comp100728_c0_seq1	721	hyperosmotic protein 21 [B	XP_001892669.1	31%	2.00E-39
comp100779_c0_seq3	363	calmodulin, isoform A [Dro	NP_523710.1	90%	2.00E-55
comp101244_c1_seq1	436	moesin/ezrin/radixin homol	XP_004931204.1	67%	5.00E-58
comp101651_c0_seq3	528	similar to Gadd45 CG11086-	XP_975522.1	47%	3.00E-22
comp101651_c0_seq4	507	similar to Gadd45 CG11086-	XP_975522.1	49%	1.00E-23
comp101729_c0_seq1	1200	DNA mismatch repair protei	XP_001375015.1	60%	1.00E-48
comp101881_c0_seq1	1408	similar to dusky-like CG15	XP_968018.2	44%	1.00E-31
comp101881_c0_seq2	910	similar to dusky-like CG15	XP_968018.2	46%	5.00E-32
comp102230_c0_seq1	934	parkin co-regulated gene p	NP_001134533.1	51%	2.00E-81
comp102374_c0_seq1	628	Voltage-dependent calcium	EFN82075.1	93%	5.00E-93
comp102374_c0_seq3	530	voltage-dependent p/q type	XP_001648697.1	67%	2.00E-60
comp102917_c0_seq2	456	mothers against decapentap	XP_004059479.1	90%	3.00E-54
comp102917_c0_seq3	456	mothers against decapentap	XP_005347842.1	93%	8.00E-57
comp103611_c0_seq1	487	similar to rac-gtp binding	XP_968845.2	99%	3.00E-45
comp103611_c0_seq3	453	similar to rac-gtp binding	XP_968845.2	99%	8.00E-72
comp103684_c0_seq1	1752	potassium voltage-gated ch	XP_001951310.2	68%	0.00E+00
comp104874_c0_seq1	1215	fruitless [Tribolium casta	NP_001157690.1	34%	1.00E-15
comp105337_c0_seq3	950	Double-strand break repair	EKC19294.1	92%	1.00E-129
comp106825_c0_seq2	377	F-box/WD-repeat protein, p	XP_002431665.1	57%	3.00E-14
comp106882_c0_seq4	464	putative voltage-gated sod	ACX44802.1	99%	4.00E-60
comp106941_c0_seq5	1457	ras-related protein Ral-a-	XP_001605517.1	36%	3.00E-103
comp106941_c0_seq6	1446	ras-related protein Ral-a-	XP_001605517.1	36%	3.00E-103
comp106941_c0_seq7	1399	ras-related protein Ral-a-	XP_001605517.1	35%	1.00E-94
comp106941_c0_seq8	1388	ras-related protein Ral-a-	XP_001605517.1	36%	1.00E-94
comp107044_c0_seq1	1283	nuclear orphan receptor TR	XP_002739506.1	34%	3.00E-42
comp107375_c0_seq1	615	E3 ubiquitin-protein ligas	XP_005175480.1	100%	5.00E-74
comp107375_c0_seq2	449	E3 ubiquitin-protein ligas	XP_004526204.1	83%	9.00E-32
comp107375_c0_seq3	417	E3 ubiquitin-protein ligas	XP_004526204.1	82%	2.00E-43
comp107536_c0_seq3	934	GI21981 [Drosophila mojave	XP_002001427.1	71%	9.00E-76
comp107822_c0_seq5	1063	Spectrin alpha chain, puta	XP_002430009.1	95%	0
comp108173_c0_seq2	1250	large conductance calcium	AF452164_1	99%	0

comp108173_c0_seq3	1249	large conductance calcium	AF452164_1	99%	0
comp108173_c0_seq4	638	large conductance calcium	AF452164_1	97%	5.00E-96
comp108173_c0_seq5	637	large conductance calcium	AF452164_1	97%	2.00E-117
comp108272_c0_seq1	1500	hypothetical protein LOC10	XP_001942708.2	99%	3.00E-158
comp108330_c0_seq2	441	cyclin dependent kinase 2	NP_001154934.1	97%	8.00E-87
comp108637_c0_seq1	4033	vascular endothelial growt	XP_006027558.1	64%	4.00E-96
comp109255_c0_seq5	375	similar to MUS81 endonucle	XP_967772.1	99%	3.00E-33
comp109408_c0_seq2	1033	tropomyosin [Periplaneta a	AAD19606.1	51%	4.00E-70
comp109408_c0_seq5	1000	tropomyosin-2 isoform 4 [B	NP_001093277.1	53%	3.00E-76
comp109408_c0_seq7	1000	tropomyosin [Periplaneta a	AAD19606.1	52%	3.00E-79
comp109408_c0_seq9	967	tropomyosin-2 isoform 4 [B	NP_001093277.1	53%	1.00E-85
comp109633_c0_seq2	613	fruitless [Tribolium casta	NP_001157690.1	38%	8.00E-45
comp109633_c0_seq3	439	male-specific transcriptio	AAV52864.1	77%	7.00E-51
comp109817_c3_seq1	935	dihydrolipoamide acetyltra	XP_001866712.1	94%	2.00E-120
comp109817_c3_seq2	872	dihydrolipoamide acetyltra	XP_001866712.1	91%	2.00E-114
comp109922_c0_seq6	459	cytochrome P-450, putative	XP_002427451.1	64%	1.00E-50
comp109959_c1_seq12	660	hypothetical protein Phum_	XP_002426736.1	81%	4.00E-55
comp109959_c1_seq13	579	hypothetical protein Phum_	XP_002426736.1	93%	1.00E-55
comp109959_c1_seq8	1023	similar to CG11071 CG11071	XP_971618.1	42%	7.00E-70
comp109959_c1_seq9	986	similar to CG11071 CG11071	XP_971618.1	44%	7.00E-70
comp110323_c0_seq1	1210	intraflagellar transport p	XP_005106145.1	99%	2.00E-147
comp110517_c0_seq1	1448	Jagged-2 precursor, putati	XP_002429076.1	98%	0.00E+00
comp111136_c0_seq2	927	casein kinase I isoform al	XP_006021125.1	39%	6.00E-30
comp111137_c0_seq3	1472	Chromosome-associated kine	XP_002423498.1	78%	9.00E-50
comp111217_c0_seq1	2821	poly(A)-specific ribonucle	XP_003690772.1	51%	6.00E-172
comp111217_c0_seq2	2682	poly(A)-specific ribonucle	XP_004057290.1	50%	2.00E-106
comp111217_c0_seq3	1958	poly(A)-specific ribonucle	XP_002918111.1	86%	9.00E-153
comp111217_c0_seq4	1819	poly(A)-specific ribonucle	XP_003707832.1	82%	5.00E-85
comp111701_c0_seq2	1402	hypothetical protein SINV_	EFZ10860.1	46%	9.00E-152
comp111748_c0_seq1	860	sperm-associated antigen 6	XP_003400399.1	99%	1.00E-118
comp111748_c0_seq2	379	sperm-associated antigen 6	XP_003492620.1	98%	1.00E-55
comp111795_c0_seq3	1688	similar to cAMP-dependent	XP_968170.1	61%	0
comp112099_c0_seq4	531	similar to thickveins CG14	XP_970678.1	35%	1.00E-23
comp112121_c0_seq2	1478	spire, putative [Pediculus	XP_002424546.1	74%	9.00E-116
comp112168_c0_seq3	668	E3 ubiquitin-protein ligas	XP_003708524.1	85%	4.00E-108

comp112168_c0_seq5	612	E3 ubiquitin-protein ligas	XP_003708524.1	93%	2.00E-108
comp112437_c1_seq1	3472	Nesprin-1 [Harpegnathos sa	EFN84145.1	42%	4.00E-142
comp112525_c0_seq1	3533	nuclear cap-binding protei	XP_003699951.1	62%	0
comp112943_c0_seq1	1823	Mitogen-activated protein	EKC18156.1	50%	7.00E-124
comp112943_c0_seq2	1812	Mitogen-activated protein	EKC18156.1	50%	5.00E-124
comp112943_c0_seq6	1419	Mitogen-activated protein	EKC18156.1	50%	9.00E-101
comp113212_c0_seq1	1298	similar to cullin [Triboli	XP_974579.1	58%	3.00E-129
comp113381_c1_seq1	419	tubulin gamma-1 chain [Fal	XP_005439501.1	57%	5.00E-41
comp113694_c0_seq2	426	class D atypical G-protein	XP_002429581.1	84%	3.00E-34
comp113806_c0_seq4	896	class D atypical G-protein	XP_002425255.1	96%	4.00E-63
comp113806_c0_seq6	833	class D atypical G-protein	XP_002425255.1	95%	3.00E-61
comp113832_c0_seq1	1769	F-box/WD repeat-containing	ETE63688.1	44%	2.00E-151
comp113832_c0_seq2	1747	F-box/WD repeat-containing	ETE63688.1	44%	8.00E-146
comp113832_c0_seq3	1190	F-box/WD repeat-containing	ETE63688.1	66%	2.00E-154
comp113832_c0_seq4	1168	F-box/WD repeat-containing	ETE63688.1	65%	9.00E-149
comp113832_c0_seq5	1023	F-box/WD repeat-containing	ETE63688.1	77%	1.00E-155
comp113956_c1_seq2	1069	Plastin-3, putative [Pedic	XP_002426666.1	60%	1.00E-126
comp114098_c0_seq10	516	similar to polybromo-1 [Tr	XP_001808258.1	100%	6.00E-61
comp114098_c0_seq7	516	similar to polybromo-1 [Tr	XP_001808258.1	70%	2.00E-28
comp114098_c0_seq8	516	similar to polybromo-1 [Tr	XP_001808258.1	83%	3.00E-32
comp114098_c0_seq9	516	similar to polybromo-1 [Tr	XP_001808258.1	87%	3.00E-57
comp114480_c1_seq1	1197	elongator complex protein,	XP_002424664.1	84%	0
comp114491_c0_seq1	792	hypothetical protein TcasG	EEZ98802.1	45%	1.00E-32
comp114491_c0_seq2	791	hypothetical protein TcasG	EEZ98802.1	57%	1.00E-42
comp114491_c0_seq3	716	hypothetical protein TcasG	EEZ98802.1	52%	7.00E-33
comp114664_c1_seq2	4250	Serine/threonine-protein k	EGI67888.1	37%	0.00E+00
comp114664_c1_seq5	4092	misshapen-like kinase 1 is	XP_003426313.1	35%	0
comp114676_c0_seq3	1465	Upstream stimulatory facto	XP_002426967.1	44%	2.00E-48
comp114676_c0_seq4	1450	Upstream stimulatory facto	XP_002426967.1	45%	2.00E-48
comp114706_c1_seq10	799	serine/threonine-protein k	XP_003696149.1	87%	9.00E-102
comp114725_c0_seq4	1523	discs large 1 [Gryllus bim	BAK23256.1	99%	0
comp114725_c0_seq6	1493	discs large 1 [Gryllus bim	BAK23256.1	99%	0
comp114771_c1_seq2	3699	p80 RT [Moloney murine leu	NP_955591.1	44%	0
comp114771_c1_seq22	596	protein gag/pol/env	0711245A	67%	8.00E-87
comp114801_c1_seq2	1580	protein argonaute-2-like [	XP_003426319.1	48%	1.00E-97

comp114801_c1_seq3	1250	argonaute 1, partial [Locu	AGO85968.1	34%	2.00E-93
comp114801_c1_seq6	1041	argonaute-1 [Nilaparvata l	AGH30326.1	64%	2.00E-69
comp114859_c0_seq4	575	Inosine-5'-monophosphate d	EFN84850.1	97%	2.00E-109
comp114927_c1_seq1	629	similar to transitional en	XP_966692.1	58%	8.00E-36
comp115090_c0_seq4	992	DNA mismatch repair protei	XP_003493495.1	83%	5.00E-120
comp115090_c0_seq9	382	mismatch repair protein Ms	AF412833_1	78%	4.00E-30
comp115171_c0_seq1	839	n6-adenosine-methyltransfe	XP_001650708.1	94%	7.00E-166
comp115171_c0_seq2	816	n6-adenosine-methyltransfe	XP_001650708.1	93%	2.00E-151
comp115200_c0_seq1	1751	fasciclin-2-like [Acyrthos	XP_001943556.2	42%	8.00E-82
comp115200_c0_seq6	544	fasciclin-2-like [Acyrthos	XP_001943556.2	91%	3.00E-39
comp115421_c0_seq10	890	hypothetical protein LOC10	XP_003427127.1	99%	3.00E-148
comp115421_c0_seq13	370	Muscleblind-like protein 2	EFN61631.1	48%	2.00E-28
comp115421_c0_seq2	1125	similar to muscleblind CG3	XP_001812946.1	67%	1.00E-172
comp115421_c0_seq3	1027	protein muscleblind-like i	XP_004929243.1	98%	7.00E-130
comp115421_c0_seq4	1022	protein muscleblind-like i	XP_004929243.1	99%	4.00E-121
comp115421_c0_seq5	973	hypothetical protein LOC10	XP_001812946.1	99%	5.00E-144
comp115421_c0_seq6	968	protein muscleblind-like i	XP_004929243.1	98%	1.00E-125
comp115421_c0_seq7	949	hypothetical protein LOC10	XP_003427127.1	99%	6.00E-145
comp115421_c0_seq8	944	protein muscleblind-like i	XP_004929243.1	98%	1.00E-126
comp115421_c0_seq9	895	hypothetical protein LOC10	XP_003427127.1	99%	1.00E-149
comp115454_c0_seq13	363	hypothetical protein LOC10	XP_003241821.1	46%	5.00E-70
comp115454_c0_seq2	1575	capicua protein, putative	XP_002423691.1	39%	6.00E-70
comp115507_c0_seq1	1775	hypothetical protein LOC10	XP_003424070.1	40%	2.00E-17
comp115507_c0_seq3	1595	hypothetical protein LOC10	XP_003424070.1	38%	5.00E-21
comp115522_c0_seq10	830	target of rapamycin [Blatt	ACH47049.1	99%	2.00E-147
comp115522_c0_seq11	829	target of rapamycin [Blatt	ACH47049.1	99%	8.00E-126
comp115568_c0_seq5	667	similar to exocyst complex	XP_966441.1	99%	3.00E-33
comp115568_c0_seq6	501	similar to exocyst complex	XP_966441.1	98%	6.00E-42
comp115598_c0_seq3	988	H/ACA ribonucleoprotein co	XP_005994798.1	25%	2.00E-26
comp115598_c0_seq4	987	H/ACA ribonucleoprotein co	XP_004529834.1	31%	4.00E-32
comp115653_c0_seq4	1065	hect E3 ubiquitin ligase,	XP_002427731.1	98%	6.00E-147
comp115778_c0_seq10	519	DNA mismatch repair protei	XP_005877146.1	90%	1.00E-57
comp115778_c0_seq11	504	DNA mismatch repair protei	XP_005222548.1	96%	4.00E-76
comp115778_c0_seq12	367	DNA mismatch repair protei	XP_006271475.1	85%	9.00E-28
comp115778_c0_seq9	529	DNA mismatch repair protei	XP_005907483.1	76%	7.00E-58

comp115855_c0_seq2	961	acetyl-CoA carboxylase, pu	XP_002429216.1	99%	4.00E-176
comp115858_c0_seq3	1591	GH21309 [Drosophila grimsh	XP_001986346.1	40%	4.00E-50
comp115858_c0_seq4	1546	GK21745 [Drosophila willis	XP_002063107.1	36%	1.00E-49
comp115968_c0_seq3	525	lingerer, putative [Pedicu	XP_002425572.1	97%	2.00E-42
comp116058_c0_seq2	2167	mutS protein homolog 5-lik	XP_005091912.1	57%	7.00E-110
comp116562_c0_seq3	564	GK17789 [Drosophila willis	XP_002068884.1	31%	3.00E-10
comp116562_c0_seq4	533	GK17789 [Drosophila willis	XP_002068884.1	33%	2.00E-10
comp116564_c0_seq5	849	ras-related protein Rap-1b	XP_396692.3	65%	3.00E-114
comp116575_c3_seq7	505	hypothetical protein KGM_1	EHJ66178.1	42%	2.00E-29
comp116719_c0_seq5	460	myosin-9, putative [Pedicu	XP_002423957.1	90%	3.00E-49
comp116789_c0_seq11	374	actin-binding protein anil	XP_001602952.2	73%	2.00E-37
comp116789_c0_seq3	820	actin-binding protein anil	XP_004535818.1	84%	2.00E-15
comp116789_c0_seq8	640	actin-binding protein anil	XP_001602952.2	43%	5.00E-36
comp116832_c1_seq7	594	DEAD box ATP-dependent RNA	XP_002424996.1	86%	2.00E-47
comp116832_c1_seq8	472	DEAD box ATP-dependent RNA	XP_002424996.1	78%	1.00E-47
comp116912_c1_seq5	688	limkain b1 (lkap) [Xenopus	CAJ83560.1	38%	7.00E-13
comp116992_c0_seq7	2025	hypothetical protein LOC10	XP_003490144.1	30%	2.00E-79
comp116992_c0_seq8	2013	hypothetical protein LOC10	XP_003490144.1	30%	2.00E-79
comp117039_c0_seq1	365	Small ubiquitin-related mo	EGI63783.1	63%	2.00E-31
comp117057_c1_seq9	956	hypothetical protein TcasG	EFA00207.1	30%	1.00E-35
comp117150_c3_seq5	707	DNA topoisomerase 2-like i	XP_001948540.2	97%	6.00E-134
comp117235_c0_seq1	390	Filamin-C [Harpegnathos sa	EFN86968.1	93%	6.00E-43
comp117462_c0_seq11	533	1-phosphatidylinositol-4,5	XP_001915213.2	99%	1.00E-29
comp117462_c0_seq12	532	1-phosphatidylinositol-4,5	XP_001915213.2	88%	1.00E-29
comp117462_c0_seq17	405	1-phosphatidylinositol-4,5	ELK30517.1	99%	8.00E-31
comp117500_c0_seq8	1053	adenomatous polyposis coli	XP_002738523.1	37%	5.00E-47
comp117500_c0_seq9	1052	adenomatous polyposis coli	XP_002738523.1	37%	2.00E-47
comp117547_c0_seq1	520	Chitooligosaccharidolytic	XP_002425212.1	99%	3.00E-63
comp117547_c0_seq2	503	beta-N-acetylglucosaminida	NP_001092298.1	82%	5.00E-63
comp117547_c0_seq3	395	Chitooligosaccharidolytic	XP_002425212.1	99%	4.00E-26
comp117548_c0_seq4	1251	tyrosine-protein phosphata	XP_003400421.1	95%	3.00E-159
comp117548_c0_seq6	1241	tyrosine-protein phosphata	XP_003400421.1	99%	0
comp117548_c0_seq7	1199	tyrosine-protein phosphata	XP_003400421.1	99%	0
comp117548_c0_seq8	1042	Receptor-type tyrosine-pro	XP_002422752.1	99%	1.00E-140
comp117620_c3_seq9	446	Pheromone receptor transcr	XP_002431247.1	95%	1.00E-49

comp117771_c0_seq1	701	daughterless, isoform A [D	NP_477189.1	37%	9.00E-37
comp117771_c0_seq16	643	Protein daughterless [Acro	EGI70903.1	75%	6.00E-56
comp117771_c0_seq17	603	Protein daughterless [Harp	EFN82122.1	67%	2.00E-55
comp117771_c0_seq18	585	Protein daughterless [Acro	EGI70903.1	68%	4.00E-56
comp117771_c0_seq20	545	Protein daughterless [Harp	EFN82122.1	58%	1.00E-55
comp117771_c0_seq21	545	protein daughterless-like	XP_001950085.2	78%	2.00E-59
comp117771_c0_seq3	941	hypothetical protein SINV_	EFZ19613.1	56%	4.00E-38
comp117771_c0_seq6	883	protein daughterless-like	ETE65907.1	41%	7.00E-44
comp117961_c0_seq1	423	trafficking kinesin-bindin	XP_003695406.1	99%	3.00E-51
comp118053_c0_seq4	1028	transient receptor potenti	XP_001950177.2	97%	3.00E-37
comp118108_c0_seq6	793	RecName: Full=Innexin inx1	Q9XYN0.1	49%	3.00E-74
comp118513_c0_seq12	395	Spectrin beta chain, putat	XP_002430498.1	100%	2.00E-21
comp118539_c0_seq10	555	huntingtin [Canis lupus fa	XP_536221.2	89%	1.00E-60
comp118539_c0_seq13	426	huntingtin-like [Apis mell	XP_001122101.2	97%	2.00E-46
comp171323_c0_seq1	562	outer host membrane [Enter	NP_040601.1	53%	2.00E-49
comp198266_c0_seq1	676	cytosolic phospholipase A2	NP_571370.1	96%	1.00E-50
comp203106_c0_seq1	651	fruitless zinc-finger A is	AFD01646.1	57%	8.00E-22
comp20530_c0_seq1	732	cell division protein FtsZ	YP_001264423.1	99%	1.00E-149
comp229426_c0_seq1	666	cAMP-dependent protein kin	XP_004929348.1	63%	7.00E-39
comp266526_c0_seq1	1104	class D atypical G-protein	XP_002425255.1	31%	7.00E-26
comp275220_c0_seq1	925	Meiotic recombination prot	XP_002429514.1	70%	1.00E-72
comp280846_c0_seq1	585	histone-lysine N-methyltra	NP_001102373.2	51%	7.00E-23
comp280846_c0_seq2	585	histone-lysine N-methyltra	NP_001102373.2	66%	5.00E-33
comp284853_c0_seq1	381	pachytene checkpoint prote	XP_003382422.1	100%	2.00E-43
comp287004_c0_seq1	927	serine/threonine-protein k	NP_035251.3	91%	1.00E-69
comp29490_c0_seq1	420	RecName: Full=Replication	P03688.1	100%	9.00E-93
comp303498_c0_seq1	639	similar to sticks and ston	XP_972469.2	90%	6.00E-95
comp304166_c0_seq1	366	cytoplasmic polyadenylatio	XP_005994168.1	47%	1.00E-18
comp307287_c0_seq1	1045	shaker cognate w, isoform	NP_722937.2	99%	0
comp315830_c0_seq1	766	hypothetical protein TcasG	EFA09839.1	99%	4.00E-107
comp319005_c0_seq1	587	RecName: Full=Ankyrin repe	Q07DV3.1	75%	2.00E-20
comp32436_c0_seq2	705	voltage-gated sodium chann	ACX44801.1	99%	1.00E-159
comp33738_c0_seq1	466	TIMELESS [Gryllus bimacula	BAJ16356.1	45%	2.00E-23
comp340062_c0_seq1	412	Dmc1 homolog [Bombyx mori]	NP_001037552.1	38%	2.00E-23
comp34556_c0_seq1	753	DNA replication licensing	XP_001850585.1	100%	1.00E-129

comp35096_c0_seq1	985	Out at first protein [Acro	EGI60768.1	83%	5.00E-98
comp35688_c0_seq1	1320	hypothetical protein KGM_0	EHJ76701.1	94%	5.00E-83
comp377050_c0_seq1	433	cell division protein FtsW	WP_004932776.1	83%	3.00E-52
comp377050_c0_seq2	432	cell division protein FtsW	ZP_10110299.1	78%	6.00E-39
comp40137_c0_seq1	1149	Prdm9 [Apodemus sylvaticus	ADA68116.1	30%	7.00E-32
comp43183_c0_seq1	1017	AGAP004311-PA [Anopheles g	XP_313585.4	73%	9.00E-90
comp433082_c0_seq1	513	DNA repair protein RAD51 h	XP_001097058.1	99%	1.00E-102
comp434554_c0_seq1	523	voltage-gated sodium chann	ACX44801.1	88%	7.00E-90
comp500877_c0_seq1	383	hypothetical protein SINV_	EFZ13804.1	63%	1.00E-23
comp51339_c0_seq1	1321	Putative helicase Mov10l1	EFN70769.1	94%	3.00E-89
comp514047_c0_seq1	506	Bardet-Biedl syndrome 2 [T	XP_002189773.1	98%	5.00E-36
comp514047_c0_seq2	506	Bardet-Biedl syndrome 2 [T	XP_002189773.1	99%	1.00E-80
comp56865_c0_seq1	606	hypothetical protein SINV_	EFZ19192.1	34%	2.00E-26
comp60966_c0_seq2	490	thymidylate synthase [Lito	ACS44779.1	99%	9.00E-91
comp621000_c0_seq1	401	similar to serine/threonin	XP_972118.2	99%	1.00E-58
comp665465_c0_seq1	393	takeout/JHBP-like protein	ABP51960.1	78%	1.00E-28
comp67596_c0_seq1	1110	mismatch repair endonuclea	XP_005721744.1	40%	1.00E-31
comp70185_c0_seq1	688	Uracil-DNA glycosylase [Cr	EKC38003.1	88%	8.00E-102
comp73426_c0_seq2	645	hypothetical protein SerAS	YP_004499171.1	64%	7.00E-67
comp73642_c0_seq1	1132	similar to sticks and ston	XP_972469.2	99%	0
comp740316_c0_seq1	437	Tail fiber protein [Entero	NP_040602.1	36%	4.00E-26
comp77254_c0_seq2	353	conserved hypothetical pro	XP_001848733.1	90%	2.00E-39
comp77468_c0_seq3	1240	Exostosin-1, putative [Ped	XP_002429885.1	90%	0
comp78460_c0_seq1	504	steroidogenic factor, puta	XP_002430380.1	64%	2.00E-37
comp78460_c0_seq2	460	steroidogenic factor, puta	CAQ57670.1	64%	2.00E-37
comp835223_c0_seq1	395	dumpy, isoform D [Drosophi	NP_001245870.1	99%	7.00E-51
comp84058_c0_seq1	808	zinc finger protein 350 [X	NP_001016767.1	83%	7.00E-12
comp84058_c0_seq2	807	zinc finger protein [Aedes	XP_001658713.1	74%	3.00E-10
comp84072_c0_seq3	407	putative dedicator of cyto	XP_003378177.1	42%	2.00E-19
comp84318_c0_seq1	495	ribosomal protein S13 [Bom	NP_001091754.1	80%	4.00E-84
comp84318_c0_seq2	494	ribosomal protein S13 [Bom	NP_001091754.1	87%	3.00E-93
comp84349_c0_seq1	403	broad-complex, isoform Z4	CBJ05860.1	73%	6.00E-42
comp85793_c0_seq1	800	homeobox protein prospero/	XP_002427668.1	93%	4.00E-35
comp86246_c0_seq3	359	shaker protein [Drosophila	CAA55519.1	45%	5.00E-31
comp86317_c0_seq1	432	ubiquitin/ribosomal protei	NP_001037372.1	83%	8.00E-49

comp87452_c0_seq2	561	Myosin-Va [Harpegnathos sa	EFN84300.1	33%	5.00E-31
comp88060_c0_seq1	1223	hypothetical protein LOC10	XP_003393831.1	89%	4.00E-81
comp90053_c0_seq1	450	centromere protein X-like	XP_003217344.1	54%	4.00E-16
comp93149_c0_seq1	656	DNA repair protein RAD51 h	XP_001509367.1	93%	1.00E-38
comp93657_c0_seq1	569	similar to H2A histone fam	XP_967411.1	52%	2.00E-45
comp93657_c0_seq2	569	similar to H2A histone fam	XP_967411.1	52%	3.00E-59
comp93820_c0_seq2	640	peptidyl-prolyl cis-trans	XP_002411993.1	43%	4.00E-31
comp94977_c0_seq2	851	Insulin-like growth factor	ELK02272.1	39%	3.00E-35
comp95468_c0_seq2	1478	TAR RNA-binding protein is	AEE36483.1	36%	4.00E-80
comp95669_c0_seq2	460	Bardet-Biedl syndrome 2 pr	XP_005107459.1	50%	3.00E-23
comp95782_c0_seq1	536	voltage-dependent calcium	XP_004536670.1	78%	2.00E-32
comp95782_c0_seq2	386	voltage-dependent calcium	XP_001943929.2	67%	1.00E-28
comp98392_c0_seq3	354	putative cornichon protein	EHJ74275.1	81%	5.00E-22
comp98675_c0_seq1	926	gliotactin [Aedes aegypti]	XP_001659685.1	94%	4.00E-155
comp99027_c0_seq1	1733	breast cancer type 2 susce	XP_003450222.1	36%	2.00E-52
comp99589_c0_seq3	875	serine/threonine-protein k	XP_623436.1	66%	4.00E-109
comp99589_c0_seq4	810	GA26044 [Drosophila pseudo	XP_002132837.1	62%	2.00E-74
